# Ethoxyquin provides neuroprotection against cisplatin-induced neurotoxicity

**DOI:** 10.1038/srep28861

**Published:** 2016-06-28

**Authors:** Jing Zhu, Valentina Alda Carozzi, Nicole Reed, Ruifa Mi, Paola Marmiroli, Guido Cavaletti, Ahmet Hoke

**Affiliations:** 1 Departments of Neurology and Neuroscience, Johns Hopkins School of Medicine, Baltimore, MD, USA; 2 Jiangsu Key Laboratory for Pharmacology and Safety Evaluation of Chinese Materia Medica, Nanjing University of Chinese Medicine, Nanjing 210023, China; 3 Experimental Neurology Unit, School of Medicine and Surgery, University of Milan-Bicocca, Monza MB, Italy; 4 Young Against Pain Group, Italy

## Abstract

Ethoxyquin was recently identified as a neuroprotective compound against toxic neuropathies and efficacy was demonstrated against paclitaxel-induced neurotoxicity *in vivo*. In this study we examined the efficacy of ethoxyquin in preventing neurotoxicity of cisplatin in rodent models of chemotherapy-induced peripheral neuropathy and explored its mechanism of action. Ethoxyquin prevented neurotoxicity of cisplatin *in vitro* in a sensory neuronal cell line and primary rat dorsal root ganglion neurons. *In vivo*, chronic co-administration of ethoxyquin partially abrogated cisplatin-induced behavioral, electrophysiological and morphological abnormalities. Furthermore, ethoxyquin did not interfere with cisplatin’s ability to induce tumor cell death in ovarian cancer cell line *in vitro* and *in vivo*. Finally, ethoxyquin reduced the levels of two client proteins (SF3B2 and ataxin-2) of a chaperone protein, heat shock protein 90 (Hsp90) when co-administered with cisplatin *in vitro*. These results implied that the neuroprotective effect of ethoxyquin is mediated through these two client proteins of Hsp90. In fact, reducing levels of SF3B2 in tissue-cultured neurons was effective against neurotoxicity of cisplatin. These findings suggest that ethoxyquin or other compounds that inhibit chaperone activity of Hsp90 and reduce levels of its client protein, SF3B2 may be developed as an adjuvant therapy to prevent neurotoxicity in cisplatin-based chemotherapy protocols.

Cisplatin (cis-diaminodichloroplatinum) was the first platinum based antineoplastic agent developed in 1970s. Since then it has been widely used in chemotherapeutic regimens for solid tumors of the ovary, skin, lung, testis, and head and neck^1^,^2^. A common side effect of cisplatin is neurotoxicity^3^,^4^ leading to chemotherapy induced peripheral neuropathy (CIPN). This dose-dependent toxic neuropathy is associated with distal paresthesias affecting hands and feet, often starting within a month of initiating cisplatin treatment and coasting after cessation of the chemotherapy. Accumulation of cis-platinum in dorsal root ganglion (DRG) neurons in the form of platinum-DNA adducts^5^ is thought to be one of the primary mechanisms of neurotoxicity. High prevalence of neurotoxicity limits chemotherapeutic efficacy of cisplatin and there are no existing drugs that can prevent development of CIPN.

Ethoxyquin (1,2-dihydro-6-ethoxy-2,2,4, trimethylqyuinoline, EQ) has been used as an antioxidant in animal feed for many years, but pharmacological use of EQ has never been evaluated in humans despite that small amounts of EQ exist in certain food items. Recently, we identified EQ as a neuroprotective compound against neurotoxicity of paclitaxel, another commonly used chemotherapy drug^6^. In this previous study, identification of EQ as a neuroprotective compound was achieved through a phenotypic screen and followed by a mechanistic study, which showed that EQ acts as an inhibitor of chaperone activity of Hsp90 reducing levels of two of its client proteins SF3B2 (a component of spliceosome complex) and ataxin-2^6^.

Since the mechanism of neurotoxicity and development of neuropathic pain differs among chemotherapeutic agents^7^, in this study, we explored the potential of EQ to provide broad neuroprotection against toxicity of a different class of chemotherapy. In addition, since neurotoxicity of cisplatin is considered to be mediated through formation of DNA-platinum adducts^8^,^9^, we examined the effect of EQ on DNA-platinum adduct formation with a new assay we developed.

## Results

### EQ prevents neurotoxicity of cisplatin *in vitro*

**Figure 1:**
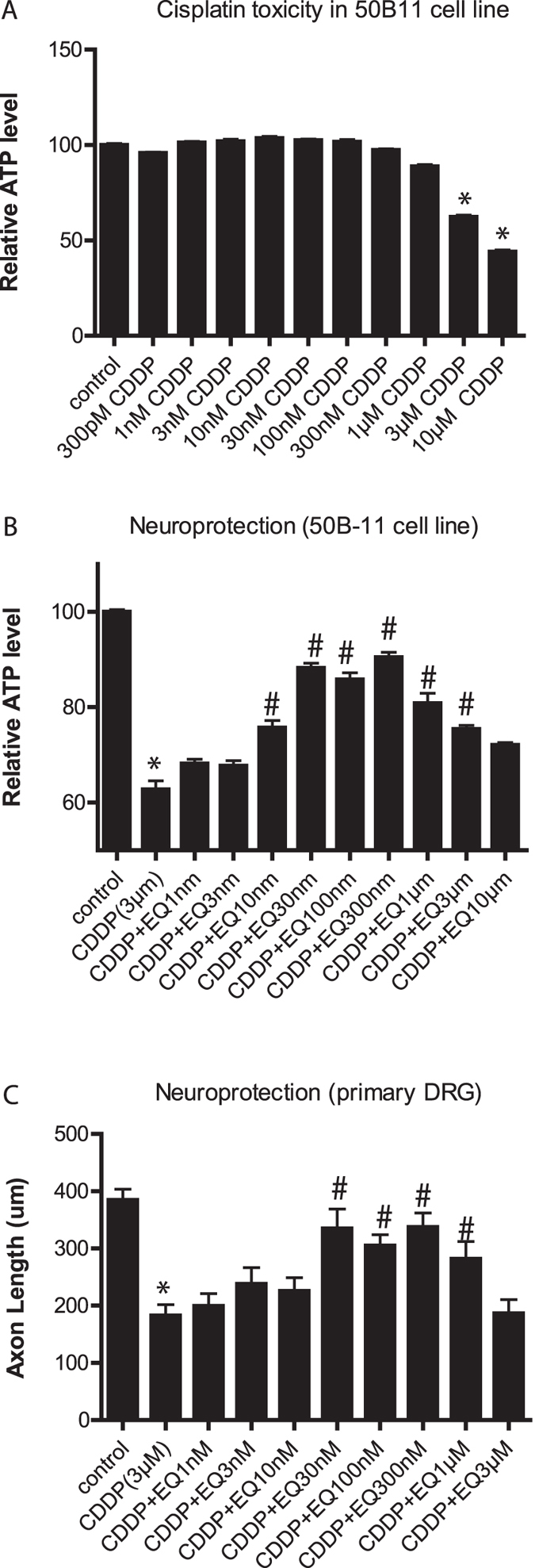
Ethoxyquin prevents cisplatin-induced neurotoxicity *in vitro*. **(A)** Differentiated 50B11 DRG neuronal cells were exposed to various concentrations of cisplatin (CDDP) for 24 hours and ATP levels were measured (n = 4–6; *p < 0.05 compared to control; error bars denote SEM). **(B)** Differentiated 50B11 DRG neuronal cells were exposed cisplatin with or without various concentrations of EQ for 24 hours and ATP levels were measured. (n = 4–6; *p < 0.05 compared to control; ^#^p < 0.05 compared to cisplatin alone; error bars denote SEM). **(C)** Established primary rat DRG neuron-Schwann cell co-cultures were exposed to cisplatin or EQ for 24 hours before fixation, staining and measurements of axon lengths. Distal axonal degeneration induced by cisplatin was partially prevented by EQ. (n = 8–10 per group; *p < 0.05 compared to control; ^#^p < 0.05 compared to CDDP alone; error bars denote SEM).

We used 50B11, an immortalized rat DRG neuronal cell line, to examine the neurotoxicity of cisplatin and the effect of EQ on its neurotoxicity by measuring cellular ATP levels. 50B11 cells were incubated with cisplatin at different concentrations, ranging from 300 pM to 10 μM. As seen in Fig. 1A, cisplatin caused 40% toxicity at 3 μM, and toxicity reached 90% at higher concentrations. When cells were exposed to cisplatin at 3 μM concentration combined with varying amounts of EQ (1 nM to 10 μM), there was consistent neuroprotection from 30 nM to 300 nM (Fig. 1B). When we repeated the experiments using primary rat DRG neuron-Schwann cell co-cultures, we found that ethoxyquin provided neuroprotection with a best efficacy range from 30 to 300 nM (Fig. 1C). It is interesting to note that higher (μM) concentrations of EQ do not provide further neuroprotection and in fact at these higher concentrations EQ loses its neuroprotective effects. This is similar to what we have observed previously against paclitaxel^6^.

### EQ prevents neurotoxicity of cisplatin in an animal model

**Figure 2:**
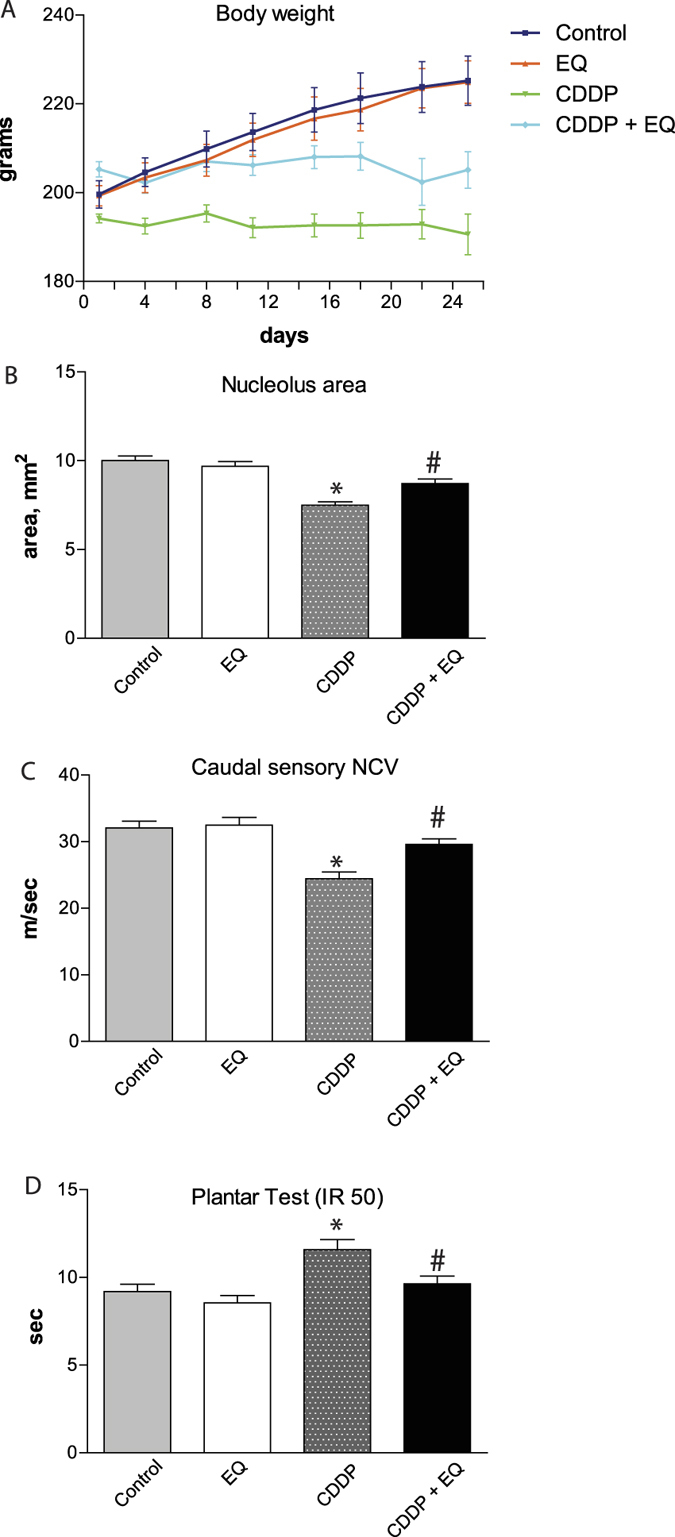
Ethoxyquin partially prevents cisplatin-induced peripheral neuropathy in a rodent model. Daily administration of EQ (75 ug/kg/d) in animals given bi-weekly injection of cisplatin did not have an effect on body weight **(A)** but partially prevented cisplatin-induced peripheral neuropathy as evaluated by morphometric measurement of the nucleolus area of DRG sensory neurons **(B)**, nerve conduction velocity in the caudal nerves **(C)**, plantar thermal withdrawal latency **(D)** (n = 8–10 per group; *p < 0.05 compared to control; ^#^p < 0.05 compared to cisplatin alone; error bars denote SEM).

Next we asked if EQ, when co-administered, could prevent neuropathy caused by cisplatin. We used a rat model that results in mild sensory neuropathy with administration of cisplatin (4 mg/kg, intravenously) twice a week for 4 weeks^10^. EQ was administered daily by intraperitoneal injection (75 μg/kg/day) either alone or with cisplatin, starting on day 1 and continued daily throughout the 4 weeks. As seen in Fig. 2A, rats treated with EQ alone or control vehicle treated rats gained weight as expected under normal physiological conditions. Both cisplatin and cisplatin/EQ treated rats did not show any weight gain suggesting that EQ did not have any appreciable effect on systemic toxicity of cisplatin. However, when we examined the neurotoxicity of cisplatin, we found a partial prevention of toxicity in pathological (nucleolus area), behavioral (thermal sensation) and electrophysiological (sensory nerve conduction velocity) outcome measures (Fig. 2B–D). Cisplatin induced significant nucleolar atrophy while the co-treatment with EQ was able to maintain physiological nucleolar sizes (Fig. 2B). Similarly cisplatin induced reduction in sensory nerve conduction velocity and thermal hypoalgesia were completely prevented by the co-administration of EQ (Fig. 2C,D, respectively).

### EQ does not block anti-cancer activity of cisplatin *in vitro* or *in vivo*

**Figure 3:**
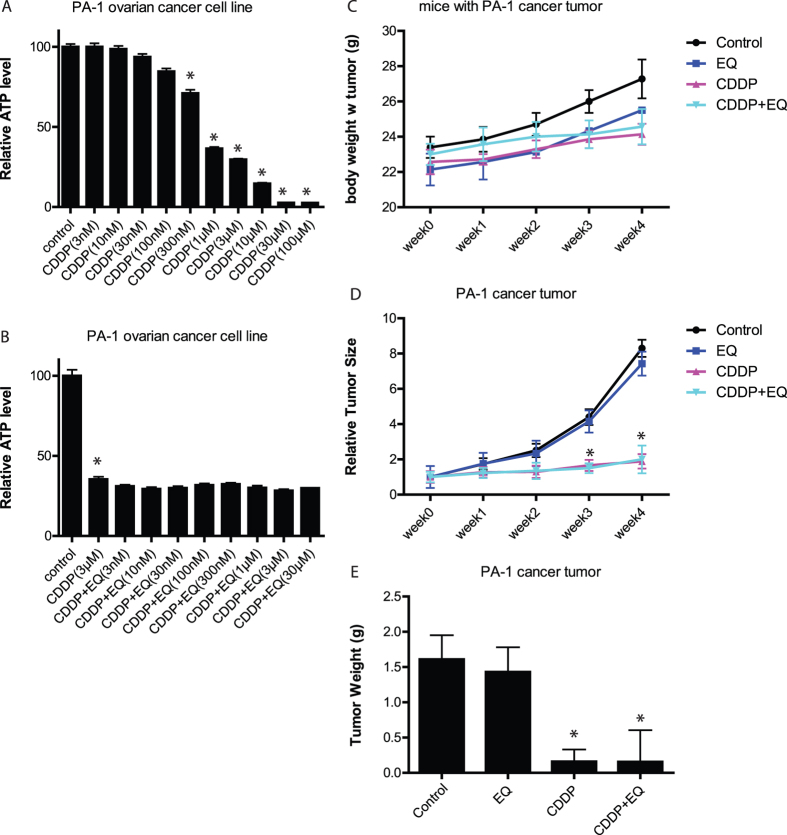
Cisplatin’s anti-tumor activity is not blocked by ethoxyquin. **(A)** When ovarian cancer cell line PA-1 was grown in culture, cisplatin reduced cell viability by 20–90%. **(B)** A wide dose range of EQ did not alter cisplatin’s ability to reduce cell viability. (n = 8–10 group; *p < 0.05 compared to control; error bars denote SEM). **(C)** After the formation of a tumor in immunodeficient mice injected with PA-1 cancer cells, cisplatin or EQ did not have any appreciable effect on body weight. In cisplatin treated mice, the tumor size **(D)** and tumor weight **(E)** were reduced but EQ did not have an effect. (n = 7 per group; *p < 0.05 compared to control; error bars denote SEM).

In order to be able to use EQ as a co-drug to prevent neurotoxicity of cisplatin, we needed to evaluate its potential impact on anti-cancer activity of cisplatin. As shown in Fig. 3A, cisplatin caused >50% cell death in PA-1 ovarian cancer cells at or above 1 μM concentration. When combined with cisplatin at 3 μM, varying doses of EQ did not have any effect on cisplatin’s ability to kill PA-1 ovarian cancer cells (Fig. 3B). When PA-1 ovarian cancer cells were injected into immune deficient mice to model tumor growth *in vivo*, there was no appreciable effect on total body weight (Fig. 3C). In this model, cisplatin was able to reduce tumor growth as measured longitudinally by tumor size (Fig. 3D) and tumor weight (Fig. 3E) at the end of the study. When co-administered with cisplatin, EQ did not have an effect on this anti-cancer activity of cisplatin (Fig. 3D,E). Tumor size remained suppressed in animals treated with cisplatin and EQ, and at the end of the study tumor weights were comparable between the animals treated with cisplatin alone and animals treated with cisplatin and EQ. In animals treated with EQ alone, the tumors grew similar to the animals treated with vehicle controls.

### Effect of EQ on DNA-platinum adducts

**Figure 4:**
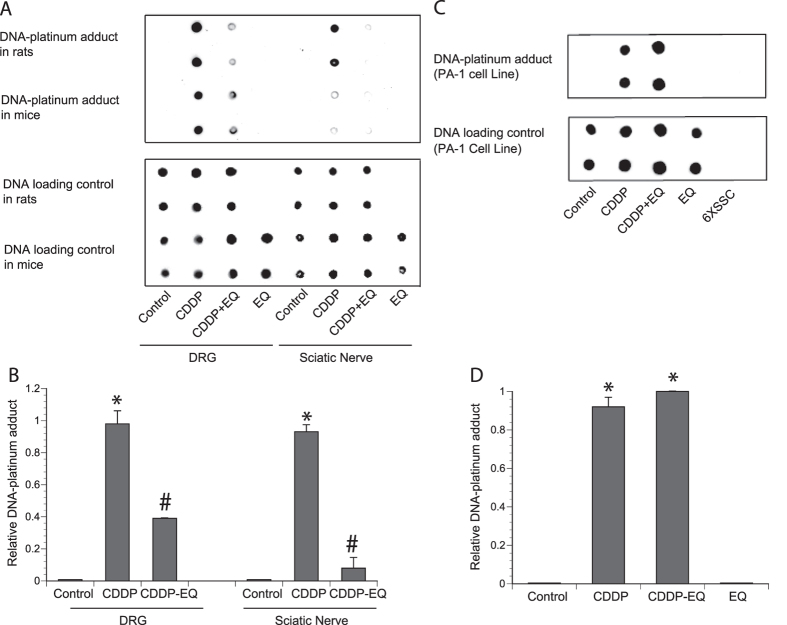
The effect of ethoxyquin on DNA-platinum adducts. **(A)** Representative dot blots of DNA-platinum adducts (in duplicates) in DNA from DRG or sciatic nerve isolated from rats and mice that were treated with CDDP, EQ or combination for four weeks (note: EQ alone group was not collected from the rats). **(B)** Densitometric ratios of dot blots from the rat samples (n = 4 per group; *p < 0.05 compared to control; *^#^p < 0.05 compared to cisplatin alone; error bars denote SD). **(C)** Representative dot blots of DNA-platinum adducts (in duplicates) in DNA isolated from ovarian cancer cell line PA-1 cultures that were treated with CDDP, EQ or combination for 24 hours. **(D)** Densitometric ratios of dot blots from the *in vitro* samples (n = 4 per group; *p < 0.05 compared to control; error bars denote SD).

Cisplatin can react with DNA and form DNA-platinum adducts, resulting in DNA damage. High levels of DNA-platinum adducts have been shown in a mouse model of cisplatin-induced peripheral neuropathy and is thought to be one of the main mechanisms of its neurotoxicity^11^. Since EQ could interfere with this mechanism, we examined levels of DNA-platinum adducts in the DRG and sciatic nerves of rat model of cisplatin induced neuropathy and mouse model of tumor growth. As seen in Fig. 4A,B, DRGs and sciatic nerves in rats and mice exposed to cisplatin had detectable levels of DNA-platinum adducts and in animals where they had exposure to both cisplatin and EQ, there was a reduction in levels of DNA-platinum adducts. Then we asked if this reduction in DNA-platinum adducts was unique to the neural tissues. When we cultured PA-1 ovarian cancer cells *in vitro* and treated them with cisplatin, there were detectable levels of DNA-platinum adduct formation but co-administration of EQ did not affect the levels of DNA-platinum adducts suggesting that EQ treatment probably would not have an impact on activity of cisplatin in tumor cells (Fig. 4C,D).

### Effect of EQ on levels of SF3B2 and ataxin-2

**Figure 5:**
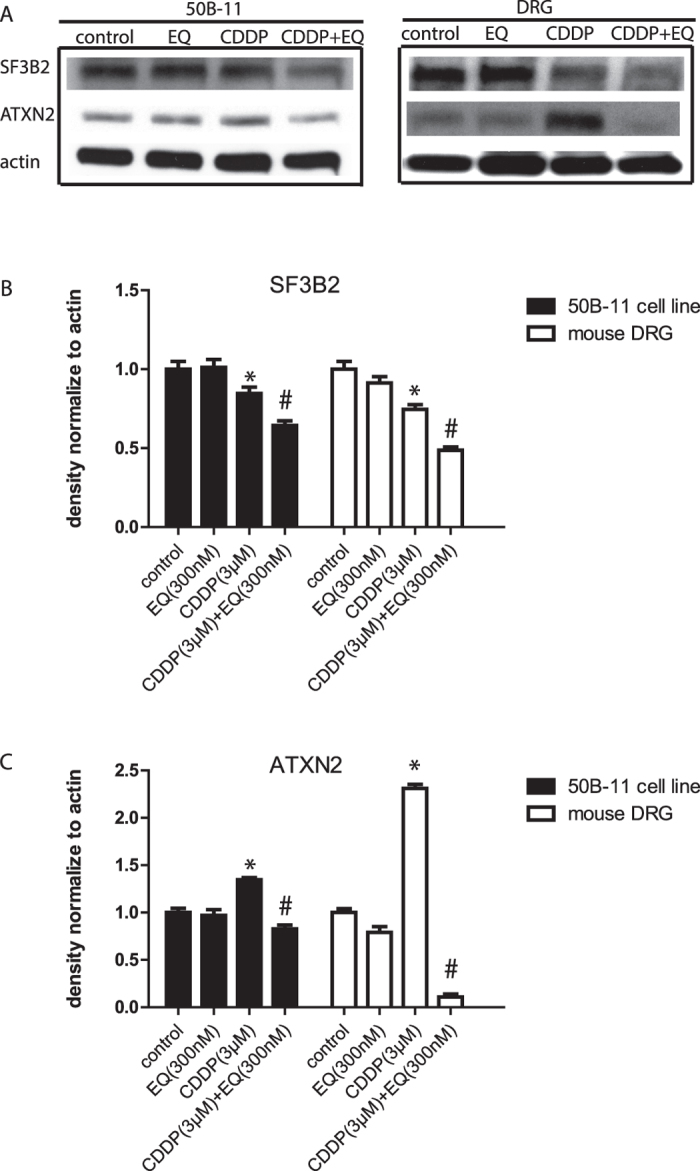
Ethoxyquin alters expression of SF3B2 and ATXN2. Representative Western blots **(A)** and densitometric ratios of SF3B2 **(B)** and ATXN2 **(C)** proteins in 50B11 DRG neuronal cultures and mouse DRG treated with CDDP and EQ (bar graphs denote means of density measurements; n = 3 per group; *p < 0.05 compared to control; ^#^p < 0.05 compared to CDDP alone; error bars denote SEM).

In our previous study where EQ was identified as a neuroprotective compound to prevent neurotoxicity of paclitaxel, we had shown that EQ inhibited the chaperone activity of Hsp90 and reduced cellular levels of two of its client proteins, SF3B2 and ataxin-2. Reducing levels of SF3B2 and ataxin-2 proteins correlated with neuroprotection against neurotoxicity of paclitaxel. In this study, we asked if the same effect was seen in cells exposed to both cisplatin and EQ and whether reduction in cellular levels of SF3B2 and ataxin-2 using RNAi were neuroprotective independent of EQ. As seen in Fig. 5, cisplatin treatment alone had opposite effects on SF3B2 and ataxin-2: cisplatin modestly reduced levels of SF3B2 but elevated levels of ataxin-2. EQ alone did not have an effect on levels of SF3B2 or ataxin-2, but when 50B11 DRG neuronal cell line were exposed to cisplatin together with EQ, there was a reduction in levels of both SF3B2 and ataxin-2. Furthermore, when we examined the levels of SF3B2 and ataxin-2 in DRGs isolated from mice treated with cisplatin and/or EQ, we found that cisplatin treatment alone resulted in lower expression of SF3B2 but higher expression of ataxin-2 levels similar to what we observed *in vitro*. Similarly, in mice treated with both cisplatin and EQ, there was a marked downregulation of ataxin-2 levels and further reduction in levels of SF3B2.

### Neuroprotection with RNAi of SF3B2 and ataxin-2

**Figure 6:**
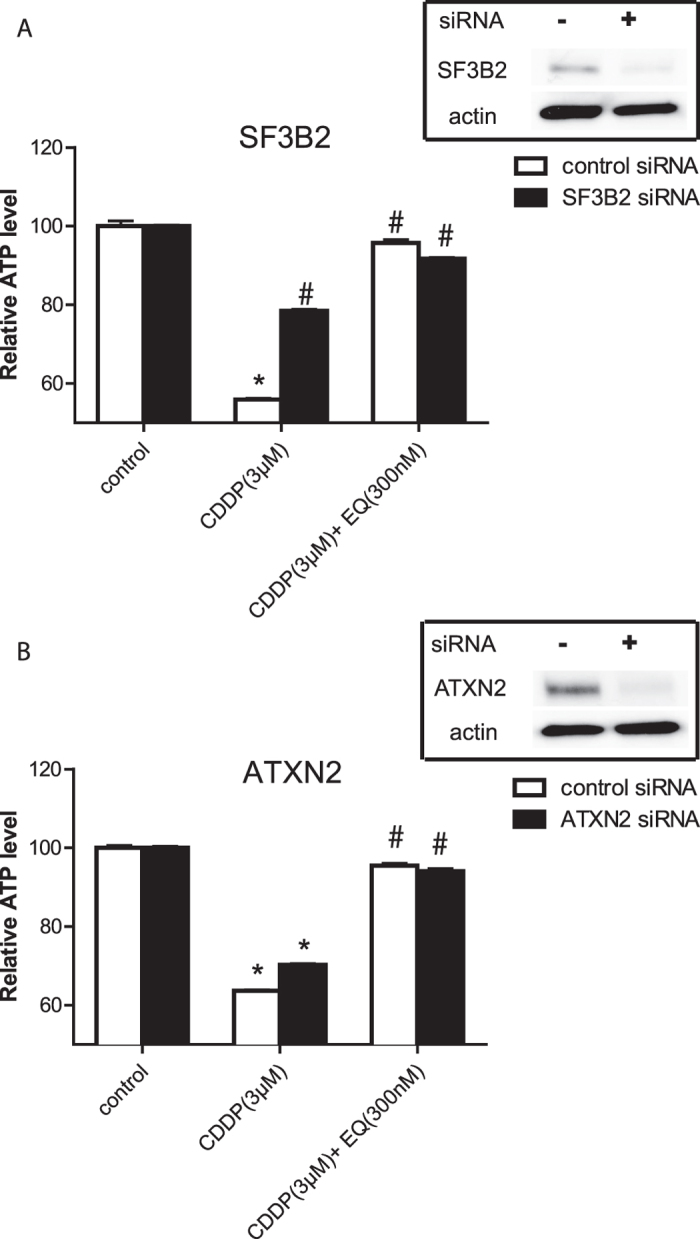
RNAi of SF3B2 but not ataxin-2 leads to loss of neuroprotection. **(A)** Reduction in SF3B2 protein level led to neuroprotection even in the absence of EQ. Addition of EQ had no further effect on neuroprotection. **(B)** In contrast, RNAi of ataxin-2 did not have any appreciable effects and addition of EQ to siRNA treated cultures still provided neuroprotection against cisplatin. (n = 8–10 per group; *p < 0.05 compared to control; ^#^p < 0.05 compared to cisplatin; error bars denote SEM).

Since reduced levels of SF3B2 and ataxin-2 in EQ-treated cells or animals were correlated with neuroprotection, we asked if reducing levels of either proteins by RNAi could result in neuroprotection in the absence of EQ. As seen in Fig. 6A, when the level of SF3B2 was reduced using siRNA, cells were resistant to toxicity of cisplatin. In fact adding EQ provided only minimal further neuroprotection. In contrast, reducing level of ataxin-2 protein alone in the cell did not provide neuroprotection (Fig. 6B). This observation suggests that reducing levels of SF3B2 may be the main mechanism by which EQ provides neuroprotection.

## Discussion

Earlier we had carried out a phenotypic drug screen and identified that ethoxyquin could inhibit neurotoxicity of paclitaxel *in vitro* and prevent peripheral neuropathy caused by paclitaxel in a mouse model. In this study we expand the potential neuroprotective activity of EQ to another class of chemotherapeutic agents and demonstrate that EQ provides neuroprotection by selectively inhibiting formation of DNA-platinum adducts in neurons but not in cancer cells. Furthermore, we show that this neuroprotection is associated with reduction in cellular levels of SF3B2 (a component of spliceosome complex) and that reducing levels of SF3B2 in neuronal cells using RNAi can result in neuroprotection against cisplatin.

Platinum containing compounds are a mainstay of many chemotherapeutic regimens but their effective use is often limited by neurotoxicity. Although the exact mechanism of neurotoxicity is unknown, accumulation of DNA-platinum adducts is thought to play an important role as this is the main mechanism of action in cancer cells. In this study we show that it is possible to dissociate the activity of cisplatin and accumulation of DNA-platinum adducts in cancer cells and in neurons. In order to detect low levels of DNA-platinum adducts in small samples, we developed a simple dot blot method to show changes in DNA-platinum adduct based on a selective and efficient antibody that recognizes DNA-platinum adducts^12^. How EQ is able to block formation of DNA-platinum adducts in neurons but not in cancer cells is unknown at this stage. It is possible that it may have a selective uptake in neuronal cells but data to support this hypothesis is lacking. Previous pharmacokinetic studies of EQ did not examine neuronal tissues^13^,^14^. Another possibility is that EQ selectively downregulates level of SF3B2 in neuronal cells but not in cancer cells. Future studies will examine this possibility.

In this study, EQ treatment resulted in reduced levels of SF3B2 and ataxin-2, two client proteins of HSP90, when neuronal cells were co-treated with cisplatin and EQ. Although inhibitors of ATPase domain of Hsp90 exhibit anti-tumor activity and several of them are in clinical trials^15^,^16^, EQ is not a typical inhibitor of Hsp90. It binds to Hsp90 and prevents the binding of its client proteins. This is similar to another inhibitor of Hsp90 with a different chemical structure (KU-32), which has been shown to be effective in diabetic neuropathy^17^. In our previous study we had identified SF3B2 and ataxin-2 as key client proteins of Hsp90 whose reduced levels were correlated with neuroprotection against paclitaxel^6^. In this study, we went one step further and demonstrated that direct reduction of cellular levels of SF3B2, but not ataxin-2, using siRNA results in neuroprotection against cisplatin toxicity. This observation suggests that SF3B2, a component of the spliceosome complex^18^, is a potential target for neuroprotection.

As part of the spliceosome complex, SF3B2 plays a potential key role in regulating alternative splicing of various mRNA species. What these key mRNA species are is unknown but upregulation of spliceosome genes is seen in various tumors^19^,^20^ suggesting that high levels of SF3B2 is associated with aberrant cell growth. Future studies comparing gene expression in neuronal cells treated with siRNA against SF3B2 and control scrambled siRNA may yield candidate genes whose expression levels (either reduced or upregulated) are important for resistance in neurons against toxicity of chemotherapeutic drugs. In fact, this pathway may even make neurons resistant to other forms of toxicity such as metabolic stress from diabetes or ischemic insults as seen in stroke. Since another Hsp90 chaperone inhibitor has therapeutic efficacy in models of diabetic neuropathy^17^, it is possible that Hsp90 chaperone inhibitors may be a broad class of drugs to provide neuroprotection. Although KU-32 has been shown to be effective in models of diabetic neuropathy, it has not been evaluated in chemotherapy neuropathy. Furthermore, although KU-32 has been shown to improve mitochondrial biogenesis, the molecular mechanism of this action is not known. It is possible that KU-32 also inhibits binding of SF3B2 and thereby reduces intracellular level of this component of the spliceosome complex.

At this point, our findings point to potential use of ethoxyquin as an adjuvant to chemotherapy regimens to prevent CIPN. Although neuroprotective drugs have been developed for many neurological diseases, including stroke, CIPN offers the ideal clinical target for neuroprotection because one could potentially co- or pre-treat the patient before neurotoxic chemotherapy drugs are given. Although EQ has never been developed as a drug, it is used in pet food as an anti-oxidant. Pharmacokinetic studies indicate oral bioavailability^13^ suggesting suitability for drug development but toxicity studies in humans or large animals do not exist. Similar to other synthetic antioxidants, it may even have antitumorigenic activity^21^. While potential genotoxicity of EQ has been raised^22^, this occurs at high concentrations of EQ (usually around 100 μM). This concentration is much higher than what is needed to bind to Hsp90 (Kd is 280 nM) and provide neuroprotection (30–300 nM). This wide margin of safety does provide a rationale to develop EQ as an adjuvant to neurotoxic chemotherapy. Further studies are needed to explore the efficacy of EQ in preventing neurotoxicity from other types of chemotherapy. In addition, drugs developed to reduce cellular levels of SF3B2 may provide an even more targeted neuroprotection.

## Methods

### Assessment of neuroprotective effects of EQ

#### *In vitro* studies

Assays examining the toxicity of cisplatin and neuroprotective effects of EQ were done using both a rat DRG neuronal cell line (50B11) and primary rat DRG neuron-Schwann cell co-cultures^6^,^23^. 50B11 rat DRG neuronal cell cultures and ATP measurements were optimized for the 96-well plate format. As described previously, 3000 cells/well were plated in 96-well plates in Neurobasal medium supplemented with 10% Penicillin Streptomycin, 10% fetal bovine serum (FBS), 0.5 mM glutamine, 1 × B-27 supplement, 0.2% glucose for 24 hours. Then the cells were differentiated with 100 μM forskolin (Sigma-Aldrich) in a culture medium with reduced serum (0.2%) for 6 hours. Varying doses of cisplatin with or without EQ were added to evaluate neurotoxicity and neuroprotection. ATP levels in each well were measured 24 hours later using ViaLight Plus kit (Lonza, catalogue # LT07-121). Vehicle media was added to serve as positive control and cisplatin alone was used as negative control. Positive and negative controls were included in each plate.

Axon lengths in primary DRG neuron-Schwann cell co-cultures were measured to evaluate the effect of EQ on cisplatin-induced neurite degeneration^6^,^23^. Dorsal root ganglia were dissected out from embryonic day 14.5 rats and dissociated before plating onto collagen coated glass coverslips. Since there was no attempt to eliminate the Schwann cells, these cultures yielded a DRG neuron-Schwann cell co-culture. Plated DRG neurons were allowed to extend neurites for 24 hour in Neurobasal medium supplemented with 50 mM PS, 0.2% fetal bovine serum (FBS), 0.5 mM glutamine, 1×B-27 supplement, 0.2% glucose, and 10 ng/mL glial cell line - derived neurotrophic factor at 37 °C. Then the DRG neurons were incubated with Cisplatin, EQ or vehicle controls for another 24 hours. Axons were delineated by fixing the cells with 4% paraformaldehyde and staining with anti-βIII-tubulin antibody (Abcam, catalogue # ab3280). A random sampling method was used to measure axon lengths in multiple fields^6^,^23^.

#### *In vivo* studies

Experiments involving animals were done after obtaining approval from the Johns Hopkins Institutional Animal Care and Use Committee and carried out according to relevant guidelines. Cisplatin (4 mg/kg) twice/week for 4 weeks was given by tail vein injections to induce peripheral neuropathy in female Wistar rats^10^. Vehicle control (saline) or ethoxyquin (75 ug/kg/day) were administered by intraperitoneal injections on a daily basis starting on day 1 with the first dose of cisplatin (n = 8 animals per group). Body weights were measured twice a week throughout the study. Thermal sensation and evoked nerve conduction studies were performed at the end of the study. DRG samples were harvested for morphometric analysis according to previously reported protocols^10^,^24^. Briefly, the antidromic nerve conduction velocity (NCV) in the tail nerve was assessed using an electromyographic apparatus (EBN Neuro, Italy) by placing recording needle electrodes (positive and negative) distally on the tail, and the stimulating needles electrodes (positive and negative) 5 and 10 cm proximally to the recording point. The “peak-to-peak” latency of the potentials was recorded and NCV calculated accordingly. The response to a noxious thermal stimulation was assessed by the plantar test using Hargreaves’s Apparatus (Ugo Basile Biological Instruments, Italy). Rats were placed into a plexiglas chamber for a 15-minute acclimation before the test. Then, a movable 40 W infrared radiant heat source was placed directly under the plantar surface of the hind paw and the time for hind paw withdrawal was monitored automatically to measure the withdrawal latency. The mean value of four repeated measures represented the nociceptive threshold. Morphometric analysis was performed on L4–L5 dorsal root ganglia obtained from rats at the end of the study (3 animals/group) and processed for resin embedding. Toluidine blue stained 1-μm-semithin sections were prepared from at least three tissue blocks for each group of animals. Somatic, nuclear, and nucleolar sizes of DRG sensory neurons were measured by using a computer assisted method (Image J software) in randomly selected sections on at least 300 DRG neurons/rat.

### Assessment of effects of EQ on cancer cells

#### *In vitro* studies

The effect of EQ on cisplatin’s antitumor activity was evaluated using an ovarian PA-1 cancer cell line (ATCC, CRL-1572) in 96-well plates. 1500 cells were plated in each well in Eagle’s Minimum Essential Medium (ATCC, Catalog No. 30-2003) supplemented with 10%FBS and incubated for 24 hours. First EQ was added followed by cisplatin and cells were cultured for another 24 hours. Cellular ATP levels as a marker of cell numbers were measured using ViaLight Plus kit (Lonza).

#### *In vivo* studies

To evaluate the impact of ethoxyquin on cisplatin’s ability to reduce tumors *in vivo*, a nude mouse model was used. Ovarian cancer cell line PA-1 tumor cells suspended in phosphate buffered saline (3 × 10^6^ cells in 150 μL) were injected subcutaneously into adult female nude mice (athymic nude mice, Cat#007850, The Jackson Lab). Once the tumor size reached 5 mm in diameter, animals were randomly assigned to receive cisplatin alone or cisplatin and ethoxyquin. Cisplatin (3.5 mg/kg/day) was given intravenously by tail vein injection once every three days for a total 8 doses and ethoxyquin (750 μg/kg/day) was administrated daily by intraperitoneal injections for 4 weeks. After four weeks, tumors (n = 7 per group) were harvested and weight and size were measured. DRGs and sciatic nerves were collected to examine the levels of DNA-platinum adducts.

### Mechanistic studies

#### Dot Blot for DNA-platinum adduct identification

2 μL of total DNA isolated from DRGs, sciatic nerves or cancer cells was blotted onto nylon membranes (Hybond-N; GE Healthcare, catalogue number RPN203N) and allowed to dry. After crosslinking the proteins by UV light, membranes we incubated in blocking solution (5% milk) for 1 hour. This was followed by incubation with anti-DNA/platinum adduct antibody (clone ICR4; EMD-Millipore, catalogue # MABE416) at 4 °C on shaker overnight. After washing the membrane 3 times for 10 minutes each, the membrane was incubated with secondary antibody (goat anti-rat IgG conjugated to HRP, Millipore, catalogue # AP136P) for 1 hour at room temperature. Then the membrane was washed again 3 times (10 minutes each), developed with ECLTM Western Blotting Detection Reagents (GE-Healthcare, catalogue # RPN2106) and exposed to X-ray film. In order to measure the total DNA loading, membrane was incubated in ethidium bromide/TBST solution until spots were visible under UV 254 nm light and images were taken with a gel documentation system.

### RNA inhibition using siRNA

Various siRNA plasmids were transfected into 50B11 DRG neuronal cells using standard protocols. Cells were cultured in 6-well plates with Neurobasal medium supplemented with 10% fetal bovine serum (FBS), 0.5 mM glutamine, 1 × B-27 supplements, and 0.2% glucose without antibiotics for 24 hours to achieve 40–60% confluence at the time of transfection. Next day, siRNA oligomers (Ambion, catalogue # s147808 for ATXN2 and catalogue # s143680 for SF3B2) were diluted by adding 1 pmol of siRNA into 250 μL Opti-MEM media (ThermoFisher Scientific, catalogue # 31985070). In a separate tube, 5 μL Lipofectamine 2000 (ThermoFisher Scientific, catalogue # 11668027) was diluted in 250 μL Opti-MEM media. The two dilutions were mixed 5 minutes later and incubated at room temperature for 20 minutes. The oligomer-Lipofectamine 2000 complexes were gently mixed with 1.5 mL Opti-MEM media and added into each well. Culture media was replaced 4 hours later with media containing blasticidin (5 μg/ml). Cells were grown for another 3 days before assaying.

### Protein isolation, Protein Electrophoresis (SDS-PAGE) and Western blotting

M-PER Mammalian Protein Extraction Reagent (Invitrogen, catalogue # 78501) mixed with protease inhibitor (ThermoFisher Scientific, catalogue # 88666) was used to extract total protein from 50B11 DRG neuronal cells, and BCA kits (ThermoFisher Scientific, catalogue # 23250) were used to measure total protein concentrations according to manufacturer’s protocols. Samples (30 μg of total protein/well) and standards (SeeBlueR Plus2 Pre-Stained Standard, Invitrogen catalogue # LC5925) were loaded onto 4–15% Mini-PROTEAN^®^ TGX™ gels (Biorad, catalogue # 456-1084) and run at 100 V for 1.5 h. After transferring onto PVDF membrane (Biorad, catalogue # 1704157) using Trans-Blot Turbo Transfer System, the membrane was blocked with 5% milk for 1 hour at room temperature and then incubated with primary antibody (anti-ATXN2 Abcam, catalogue # ab72263 or anti-SF3B2 Millipore, catalogue # ABE626) at 1:1000 dilution at 4 °C overnight. After three washes, the membrane was incubated with the secondary antibody at 1:1000 dilution at room temperature for 1 hour and then developed with ECLTM Western Blotting Detection Reagents (GE-Healthcare, catalogue # RPN2106).

### Statistical analysis

All experiments involving cells were done in triplicates and repeated at least 2–3 times. The *in vivo* studies were carried out with n = 4–8 in each group. ANOVA with correction for multiple comparisons was used for statistical analysis.

## Additional Information

**How to cite this article**: Zhu, J. *et al*. Ethoxyquin provides neuroprotection against cisplatin-induced neurotoxicity. *Sci. Rep.* **6**, 28861; doi: 10.1038/srep28861 (2016).
